# The complexity of interpreting genomic data in patients with acute myeloid leukemia

**DOI:** 10.1038/bcj.2016.115

**Published:** 2016-12-16

**Authors:** A Nazha, A Zarzour, K Al-Issa, T Radivoyevitch, H E Carraway, C M Hirsch, B Przychodzen, B J Patel, M Clemente, S R Sanikommu, M Kalaycio, J P Maciejewski, M A Sekeres

**Affiliations:** 1 Leukemia Program and Department of Translational Hematology and Oncology Research, Cleveland Clinic, Cleveland, OH, USA

## Abstract

Acute myeloid leukemia (AML) is a heterogeneous neoplasm characterized by the accumulation of complex genetic alterations responsible for the initiation and progression of the disease. Translating genomic information into clinical practice remained challenging with conflicting results regarding the impact of certain mutations on disease phenotype and overall survival (OS) especially when clinical variables are controlled for when interpreting the result. We sequenced the coding region for 62 genes in 468 patients with secondary AML (sAML) and primary AML (pAML). Overall, mutations in *FLT3, DNMT3A, NPM1* and *IDH2* were more specific for pAML whereas *UTAF1, STAG2, BCORL1, BCOR, EZH2, JAK2, CBL, PRPF8, SF3B1, ASXL1 and DHX29* were more specific for sAML. However, in multivariate analysis that included clinical variables, only *FLT3* and *DNMT3A* remained specific for pAML and *EZH2, BCOR, SF3B1* and *ASXL1* for sAML. When the impact of mutations on OS was evaluated in the entire cohort, mutations in *DNMT3A*, *PRPF8*, *ASXL1*, *CBL EZH2* and *TP53* had a negative impact on OS; no mutation impacted OS favorably; however, in a cox multivariate analysis that included clinical data, mutations in *DNMT3A*, *ASXL1*, *CBL*, *EZH2* and *TP53* became significant. Thus, controlling for clinical variables is important when interpreting genomic data in AML.

## Introduction

Acute myeloid leukemia (AML) is a heterogeneous clonal disorder characterized by the acquisition of chromosomal abnormalities and somatic mutations that drive disease phenotype, progression, and resistance to therapies.^1, 2, 3^ In the last four decades, the discovery of chromosomal abnormalities such as balanced translocations and inversions has illuminated the pathogenesis of AML and confirmed the genetic basis of the disease. Since then, cytogenetic information, along with other clinical variables such as age, disease phenotype (primary AML (pAML) vs secondary AML (sAML), and white blood cell count (WBC) at diagnosis have been used to risk stratify patients.^3, 4^ Approximately 50% of AML patients have normal karyotype (NK) without any evidence of the structural abnormalities that have subsequently been detected by higher resolution technologies such as high-density comparative genomic hybridization or single-nucleotide polymorphism arrays.^5, 6, 7^

Advances in genomic technologies have increasingly highlighted the remarkable complexity of genetic and epigenetic alterations in AML.^1, 2, 3^ Whole genome sequencing studies have identified at least one driver mutation in almost every sample from AML patients, with an average of ~13 mutations per sample.^1^ Some mutations, such as *NPM1, FLT3, CEBPA* and *DNMT3A,* are more common and have been shown to impact overall survival (OS) whereas other mutations, such as *IDH1, IDH2* and *TET2,* occur in a lower frequencies without a clear impact on OS.^8^ Further, other mutations, such as *SF3B1, SRSF2, U2AF1, ZRSR2, ASXL1, EZH2, BOCR* and *STAG2,* occur more specifically in patients with sAML compared with pAML, and can be used to define disease phenotype.^9^ Whether the specificity of these mutations is retained in all subtypes of AML, such as in patients with complex karyotype or unfavorable risk cytogenetics, has not been established. Further, controversies regarding the impact of somatic mutations on disease phenotype and OS may be related to several factors, including a small sample size in some studies, a small number of genes tested in a given panel, and the lack of careful evaluation of the impact of these mutations on outcome and disease phenotype in the setting of clinical variables such as age, cytogenetics and WBC, that inform prognosis and determination of therapeutic options. Further, some of these studies only included younger patients who received intensive chemotherapy and the application of the results of these studies in older adults who are not eligible to receive such therapy is not established.

In this study, we investigated the interplay between genomic and clinical information in a large cohort of patients with pAML and sAML using a genomic panel of the most recurrent somatic mutations in myeloid malignancies.

## Methods

### Patients

Clinical and mutational data for patients diagnosed with sAML and pAML according to 2008 World Health Organization criteria and treated at Cleveland Clinic between 1–2003 and 1–2013 were included.^10^ sAML was defined by histological interpretation of bone marrow biopsy specimens in conjunction with documentation of antecedent myelodysplastic syndromes, aplastic anemia, myelproliferative neoplasm or chronic myelomonocytic leukemia, by experienced hematopathologists not associated with this study. The pAML cohort consisted of 79 patients with complete mutational and clinical data that were randomly selected from our samples database and 168 patients from TCGA atlas (publicly available data).^1^ Patients with AML and *t*(15,17), *t*(8;21)(q22;q22), *t*(16,16) and inv(16)(p13q22) were excluded since the number of cases was not enough to derive a meaningful conclusions.

Conventional cytogenetic analyses were performed on bone marrow samples obtained at diagnosis by culturing bone marrow cells for 24–48 h using standard techniques. An abnormality was considered clonal when at least 2 metaphases had the same abnormalities, in accordance with the International System of Human Cytogenetic Nomenclature (ISCN 2005).^11^ Cytogenetic risk grouping was per CALGB/Alliance criteria.^4^

All samples and clinical data were collected with patient consent and this study was performed under an Institutional Review Board approved protocol in accordance with the Declaration of Helsinki.

### Sample processing, DNA sequencing and mutational analysis

DNA was extracted from peripheral blood or bone marrow mononuclear cells in blood samples that were stored at the Stem Cell Tissue Bank at the Cleveland Clinic. Direct sequencing was performed on coding exons of 62 genes (Supplementary Data) using Illumina TrueSeq Custom Amplicon kit. For germ-line confirmation, mutations were analyzed in non-clonal CD3þ cells whenever DNA was available. Bidirectional sequencing was performed by standard techniques using an ABI 3730xl DNA analyzer (Applied Biosystems, Foster City, CA, USA). Putative variants were extracted using GATK3.3 pipeline, following recommended best practices for variant discovery. Variants with at least 10 positive reads and variate allelic frequency of 5% were prioritized for further processing and annotation. VCF files generated were used as an input for Annovar and were annotated with multiple databases (dbSNP138, COSMIC, ExacDb). Variants found in ExacDb with allelic frequency >0.0001 were excluded. Mutations in *NPM1* and *CEBPA*, *FLT3* were also tested using standard methods. The sequencing method of the pAML cohort from TCGA database is described previously.^1^

### Treatment

Patients were not treated uniformly since our cohort included elderly patients who were not eligible to receive intensive chemotherapy or allogeneic stem cell transplant. A total of 160 patients received standard induction chemotherapy with cytarabine × 7 days +3 days of anthracycline, 53 received a hypomethylating agent +/− combination, 26 were treated on a clinical trial, 19 received low dose cytarabine, 42 received other treatment modalities (such as hydroxyurea and supportive care only) and 168 TCGA patients whom their treatments was not reported in the database.^1^

### Statistical analysis

Continuous and categorical variables were compared using Wilcoxon rank sum test and Fisher's exact test. OS was calculated from the date of diagnosis to date of last follow-up or death. A logistic regression and Cox regression multivariate analyses that included all clinical variables and significant mutations were used whichever appropriate to compare the mutation distribution and the impact of mutations on OS between pAML and sAML, respectively. *P-*values were two-sided and considered significant at <0.05. All analyses were conducted using R package language.

## Results

### Patient characteristics

A total of 468 patients were included in the final analysis, 247 with pAML and 221 with sAML (Table 1). The median age for the entire cohort was 64 years (range, 18–100) and 222 patients (47.4%) had NK. Compared with patients with pAML, those with sAML were older (68 vs 60 years, *P*<0.001), had a lower WBC at presentation (3.85 vs 13 × 10^9^ g/l, *P*<0.001), less likely to have NK cytogenetics (35.7 vs 57.9%, *P*<0.001), and more likely to have unfavorable cytogenetics (36.7 vs 23.9%, *P*=0.002), respectively (Table 1). The median follow-up for the entire cohort was 18.6 months (range, 0–114.1).

### Mutation distribution in primary versus secondary AML

**Figure 1:**
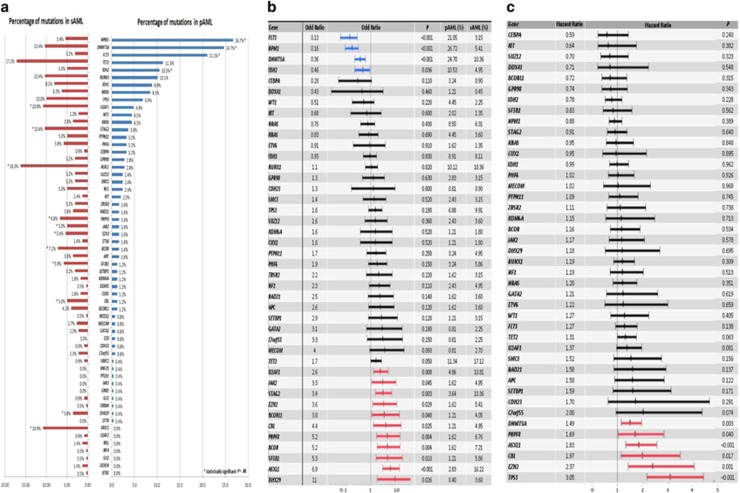
(**a**) Mutation distribution between primary and secondary AML. (**b**) Association between individual mutated genes and clinically defined secondary and primary AML as described by odds ratio on a log10 scale. (**c**) Impact of individual genes on OS as described by hazard ratio.

An average of two mutations/sample was observed (range, 0–18). Comparing the genetic landscape between pAML and sAML, distinct genomic alterations specific for each phenotype were observed (Figure 1a). Overall, mutations in *FLT3, DNMT3A, NPM1* and *IDH2* were more specific for pAML whereas 11 mutations (in *UTAF1, STAG2, BCORL1, BCOR, EZH2, JAK2, CBL, PRPF8, SF3B1, ASXL1 and DHX29)* were more specific for sAML (Figures 1a and b, Supplementary Data).

**Figure 2:**
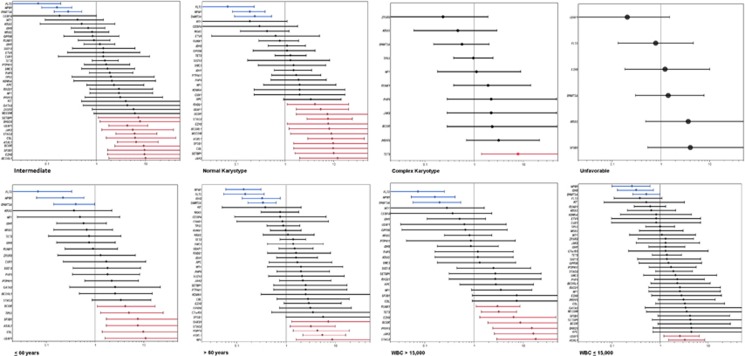
Association of individual mutations with each clinical subtype of AML defined by age, cytogenetics and WBC as described by odds ratio on a log10 scale. Blue indicates mutations that are >95% specific for primary AML and red indicates mutations that are >95% specific for secondary AML.

When the analysis was restricted to distinct cytogenetic subgroups, a different spectrum of mutations was found. In patients with NK, *FLT3, DNMT3A* and *NPM1* remained specific for pAML, but only *UTAF1, STAG2, BCORL1, BCOR, EZH2, JAK2, CBL, SF3B1* and *ASXL1* retained their specificity for sAML, with mutations in *MECOM*, *SETBP1* and *RAD21* becoming significant for the sAML phenotype (Figure 2, Supplementary Data). Similarly, mutations specific to each AML phenotype differed in patients with complex karyotype, and in those with intermediate risk and unfavorable risk cytogenentics (Figure 2, Supplementary Data).

Similar results were obtained when other clinical variables, such as age (⩽60 years vs >60 years) and WBC (⩽15 000 vs >15 000) were taken into account (Figure 2, Supplementary Data).

In a logistic regression multivariate analysis that included clinical and mutational variables, only two mutations—*FLT3* and *DNMT3A*—remained specific for pAML, whereas only four mutations—*EZH2, BCOR, SF3B1* and *ASXL1*—were specific for sAML (Table 2).

### Impact of mutations on overall survival

The median OS for patients with sAML was shorter compared with those with pAML (7.4 (range, 0–82.2) vs 11.2 (range, 0–114.1) months, respectively, *P* 0.001).

When the impact of mutations on OS was evaluated in the entire cohort, mutations in *DNMT3A* (hazard ratio (HR), 1.49, *P*.003), *PRPF8* (HR, 1.69, *P*.04), *ASXL1* (HR, 1.83, *P*<0.001), *CBL* (HR, 1.97, *P*.017), *EZH2* (HR, 2.37, *P*.001) and *TP53* (HR, 3.05, *P*<0.001) had a deleterious effect on OS, and no mutation was associated with improved OS (Figure A–C, Supplementary Data).

**Figure 3:**
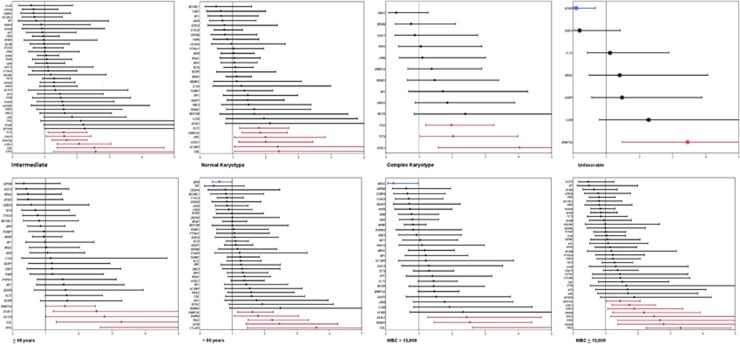
Impact of each mutation on OS in each clinical subtype of AML defined by age, cytogenetics and WBC as described by HR on a log10 scale. Blue indicates mutations that have positive impact on OS and red indicates mutations that have negative impact on OS.

When cytogenetic grouping was taken into account, different mutations had a different impact on OS (Figure 3, Supplementary Data). In patients with NK, mutations in *FLT3*, *DNMT3A*, *APC*, *ASXL1*, *SETBP1* and *CBL* had a negative impact on OS, whereas only DNMT3A mutations had a negative impact on OS in patients with unfavorable cytogenetics (Figure 3). Interestingly, mutations in *NPM1* and *CEBPA* lost their favorable impact on OS in patients with NK, likely because the analysis included patients with both pAML and sAML. Similarly, the impact of mutations on OS changed in patients ⩽60 year-old vs >60 and patients with WBC ⩽15000 vs >15000 (Figure 3, Supplementary Data).

In a cox multivariate analyses that included clinical and mutational data, only mutations in *DNMT3A*, *ASXL1*, *EZH2* and *TP53* retained their prognostic impact (Table 3). However, when the analysis was restricted to the patients with available treatment data and treatment history (intensive vs non-intensive) was added to the multivariate analysis, all mutations retained their prognostic impact on OS (Supplementary Data) suggesting that intensive chemotherapy did not overcome the negative impact of these mutations on OS.

## Discussion

AML is a heterogeneous clonal neoplasm characterized by complex genomic alterations that drive disease progression and resistance to therapy. Traditionally, sAML had been defined in part by the existence of an antecedent hematologic disorder such as myelodysplastic syndromes or myelproliferative neoplasm, whereas pAML arises *de novo*. Genomic discoveries have revealed the extent of genetic heterogenecity in AML, and have enabled better risk stratification, particularly in patients with NK. Further, certain somatic mutations are highly specific for disease phenotype (pAML vs sAML) and can in some cases define disease ontogeny, distinguishing sAML from pAML even in the absence of a confirmed preceding hematologic condition. Genomic abnormalities cannot be used in a vacuum, however, as been highlighted by divergent results regarding the impact of some mutations on OS in a subgroup of patients with AML, and the lack of reproducibility of several models that were developed to risk stratify AML patients. This raises the issue of the optimal way to interpret genomic information in AML in conjunction with traditional risk stratification tools that use clinical variables, such as age, conventional cytogenetics and treatment intensity especially when the analysis include older adults with AML who are not eligible to receive intensive chemotherapy.

In this study, we explored the interplay of established prognostic clinical variables and somatic mutations that are associated with disease phenotype and prognosis. We identified 11 mutations that are highly specific for sAML and four specific for pAML. These mutations were largely consistent with prior reports.^9^ However, when we investigated the specificity of these mutations to define disease phenotype (sAML vs pAML) in AML subtypes characterized by age, conventional cytogenetics and WBC at presentation the specificity of the mutations was lost. For example, in patients with a complex karytope at presentation, none of the somatic mutations were specific for either sAML or pAML, suggesting morphological assessment of the bone marrow biopsy by experienced hematopathologist and a history of an antecedent hematologic disorder is still needed to define disease phenotype. Moreover, when mutations were combined with clinical variables in a logistic regression multivariate analysis, only four mutations (*EZH2, BCOR, SF3B1* and *ASXL1)* remained specific for sAML whereas three mutations (*FLT3*, *NPM1* and *DNMT3A)* remained specific from pAML. In a study of 194 patients with secondary and therapy-related AML, Lindsley *et al.*^9^ showed that the presence of mutations in *SRSF2, SF3B1, U2AF1, ZRSR2, ASXL1*, *EZH2, BCOR or STAG2* were >95% specific for the diagnosis of sAML, and only *NPM1* mutations were specific for pAML. Incorporation of clinical variables, and inherent differences in patients enrolled in a formal clinical trial vs capture of all-comers, likely contributed to differences between the studies.

When we investigated the impact of recurrent somatic mutation on OS in the entire group, including patients with pAML and sAML, we found that six mutations (*DNMT3A*, *PRPF8*, *ASXL1, CBL, EZH2* and *TP53)* had a negative impact on OS, and none had a favorable effect. Similarly, the impact of mutations on OS changed when clinical variables were taken into account. In a Cox regression multivariate analysis that included significant mutations and clinical variables, only four mutations (*DNMT3A*, *ASXL1*, *EZH2* and *TP53)* retained their negative impact on OS even when treatment intensity was added as a covariate. This suggests that treatment with intensive chemotherapy may not overcome the negative impact of these mutations.

Prior evidence suggests that the impact of mutations on OS differ in subgroups of patients with AML. For example, *NPM1* mutations have a positive impact on OS in patients with NK AML, whereas their impact on OS is lost in patients with unfavorable risk cytogenetics. Similarly, *FLT3-ITD* mutations have a negative impact on OS in patients with NK, but their impact on survival is lost in patients with unfavorable risk cytogenetics. The impact of multiple mutations on overall outcome differ between younger and elder patients with AML. To add to the complexity of interpreting this information, mutations occur in the context of other mutations, which may affect outcome. For example, Patel *et al.*^12^ showed that *NPM1* mutations have a positive impact on OS only when they occur with *IDH1* or *IDH2* mutations and in the absence of *FLT3-ITD* mutations. Although the authors developed a genomic model that could be used to risk stratify patients with pAML a subsequent study could not validate it in a cohort of patients treated at a single institution.^13^ To date, there is no accepted and validated model that includes mutational data, and most clinical decisions are based on clinical variables along with only three mutations: *NPM1*, *FLT-3ITD* and *CEBPa* in patients with NK. This may evolve as novel methods such as machine learning and the ability to take advantage of large sets of databases are explored in the future.

In conclusion, clear genomic variations exist between sAML and pAML. The interpretation of genomic information should take into account clinical information along with coexistent mutations. A novel method to incorporate all this information is needed to further accurately predict AML phenotype and prognosis.

## Figures and Tables

**Table 1:** Patient characteristics

*Parameter*	*Total no. (range/%)*	*pAML no. (range/%)*	*sAML no. (range/%)*	P-*value*
Number of patients	468	247	221	
Median age, years	64 (18–100)	60 (18–93)	68 (23–100)	<0.001
** **Age>60	292 (62.4%)	125 (50.6%)	167 (75.6%)	<0.001
*Gender*				
** **Male	268 (57.3%)	131 (53%)	137 (62%)	0.05
** **Female	200 (42.7%)	116 (47%)	84 (38%)	0.05
*Clinical variables*				
** **Median WBCx10^9^/l	7.3 (0.22–387)	13 (0.5–298)	3.85 (0.22–387)	<0.001
** **WBC>15 000	171 (36%)	121 (49%)	50 (23%)	<0.001
** **Median hemoglobin, g/dl	9.1 (2.7–14)	9 (4.6–14)	9.3 (2.7–13.8)	0.31
** **Median plateletsx10^9^/l	52 (1–617)	53 (6–351)	50 (1–617)	0.21
*Cytogenetic grouping*				
** **Intermediate	300 (64.1%)	169 (68.4%)	131 (59.3%)	0.04
** **Normal karyotype	222 (47.4%)	143 (57.9%)	79 (35.7%)	
** **Unfavorable	140 (29.2%)	59 (23.9%)	81 (36.7%)	0.002
** **Complex karyotype	79 (16.9%)	32 (13%)	47 (21.3%)	
** **−5/del5q	15 (3.2%)	5 (2%)	10 (4.5%)	
** **−7/del7q	17 (3.6%)	10 (4%)	7 (3.2%)	
** **+8	29 (6.2%)	12 (4.9%)	17 (7.7%)	
** **Not available	28 (6%)	19 (7.7%)	9 (4%)	

Abbreviations: pAML, primary acute myeloid leukemia; sAML, secondary acute myeloid leukemia; WBC, white blood cell count.

**Table 2:** Multivariate logistic regression analysis comparing primary to secondary AML

*Parameter*	*Odd ratio*	*Confidence interval*	P*-value*
Age (⩽60 vs >60)	2.80	1.68–4.64	<0.001
Cytogenetic analysis (normal karyotype vs others)	2.14	1.10–4.16	0.026
Cytogenetic analysis (normal karyotype vs others)	1.64	0.90–2.97	0.105
Cytogenetic analysis (normal karyotype vs others)	1.27	0.56–2.84	0.566
WBC (⩽15 000 vs >15 000)	0.50	0.30–0.85	0.011
*FLT3*	0.17	0.05–0.57	0.004
*NPM1*	0.47	0.21–1.04	0.062
*DNMT3A*	0.33	0.16–0.67	0.002
*EZH2*	5.35	1.15–25.03	0.033
*BCOR*	7.40	2.01–27.32	0.003
*SF3B1*	7.06	1.69–29.44	0.007
*ASXL1*	3.77	1.44–9.91	0.007

Abbreviations: AML, acute myeloid leukemia; WBC, white blood cell count.

**Table 3:** Multivariate Cox regression analysis comparing primary to secondary AML

*Parameter*	*Hazard ratio*	*95% Confidence interval*	P-*value*
Diagnosis (primary vs secondary AML)	1.27	1.00–1.62	0.051
Age (⩽60 vs>60)	2.19	1.70–2.81	<0.001
Cytogenetic analysis (normal karyotype vs others)	1.00	0.74–1.35	0.992
Cytogenetic analysis (normal karyotype vs others)	2.35	1.71–3.24	<0.001
Cytogenetic analysis (normal karyotype vs others)	1.85	1.25–2.74	0.002
WBC (⩽15 000 vs >15 000)	1.44	1.12–1.84	0.004
*DNMT3A*	1.95	1.46–2.59	<0.001
*ASXL1*	1.72	1.19–2.48	0.004
*EZH2*	2.18	1.20–3.97	0.011
*TP53*	2.23	1.47–3.38	<0.001

Abbreviations: AML, acute myeloid leukemia; WBC, white blood cell count.
