# Supplementary material for: The complexity of interpreting genomic data in patients with acute myeloid leukemia
Source: Blood Cancer J. 2016 Dec 16;6(12):e510–. doi: 10.1038/bcj.2016.115 (PMC5223150; doi:10.1038/bcj.2016.115)
Supplement: Supplementary Material [file bcj2016115x1.docx]

**Table S1**: Primary AML vs. secondary AML logistic regression (all cases, mutations only)

| Gene | Odd Ratio | 95% confidence interval | | *P* |
| --- | --- | --- | --- | --- |
| *DHX29* | 11 | 1.9-200 | .03 | |
| *ASXL1* | 6.9 | 3.2-17 | <.001 | |
| *SF3B1* | 5.3 | 1.7-23 | .01 | |
| *BCOR* | 5.2 | 1.9-18 | <.001 | |
| *PRPF8* | 5.2 | 1.8-18 | <.001 | |
| *CBL* | 4.4 | 1.3-20 | .03 | |
| *BCORL1* | 3.8 | 1.1-17 | .05 | |
| *EZH2* | 3.6 | 1.2-13 | .03 | |
| *STAG2* | 3.4 | 1.6-7.9 | <.001 | |
| *JAK2* | 3.3 | 1.1-12 | .05 | |
| *U2AF1* | 2.6 | 1.3-5.6 | .01 | |
| *TET2* | 1.7 | 1-2.9 | .05 | |
| *MECOM* | 4 | 0.9-27 | .09 | |
| *C7orf55* | 3.3 | 0.71-23 | .15 | |
| *GATA2* | 3.1 | 0.66-22 | .18 | |
| *SETBP1* | 2.9 | 0.8-14 | .12 | |
| *APC* | 2.6 | 0.82-10 | .12 | |
| *RAD21* | 2.5 | 0.78-9.5 | .14 | |
| *NF1* | 2.3 | 0.86-6.8 | .11 | |
| *ZRSR2* | 2.2 | 0.65-8.4 | .22 | |
| *PHF6* | 1.9 | 0.8-5 | .15 | |
| *PTPN11* | 1.7 | 0.68-4.5 | .25 | |
| *CUX1* | 1.6 | 0.36-8.4 | .52 | |
| *KDM6A* | 1.6 | 0.36-8.4 | .52 | |
| *SUZ12* | 1.6 | 0.56-5.1 | .36 | |
| *TP53* | 1.6 | 0.81-3.1 | .19 | |
| *SMC3* | 1.4 | 0.47-4.5 | .52 | |
| *CDH23* | 1.3 | 0.15-11 | .80 | |
| *GPR98* | 1.3 | 0.44-3.9 | .63 | |
| *RUNX1* | 1.1 | 0.59-2 | .82 | |
| *IDH1* | 0.93 | 0.48-1.8 | .83 | |
| *ETV6* | 0.91 | 0.18-4.2 | .91 | |
| *KRAS* | 0.83 | 0.31-2.1 | .69 | |
| *NRAS* | 0.76 | 0.37-1.5 | .43 | |
| *KIT* | 0.68 | 0.14-2.8 | .60 | |
| *WT1* | 0.51 | 0.16-1.4 | .22 | |
| *DDX41* | 0.43 | 0.02-3.4 | .46 | |
| *CEBPA* | 0.28 | 0.04-1.1 | .11 | |
| *IDH2* | 0.46 | 0.21-0.93 | .04 | |
| *DNMT3A* | 0.36 | 0.21-0.6 | <.001 | |
| *NPM1* | 0.16 | 0.08-0.3 | <.001 | |
| *FLT3* | 0.13 | 0.05-0.27 | <.001 | |

**Table S2**: Primary AML vs. secondary AML logistic regression (Normal Karyotype cases , mutations only)

| Gene | OR | CI | P |
| --- | --- | --- | --- |
| *FLT3* | 0.067 | 0.01-0.23 | <.001 |
| *NPM1* | 0.19 | 0.08-0.39 | <.001 |
| *WT1* | 0.19 | 0.01-1.1 | .12 |
| *DNMT3A* | 0.24 | 0.09-0.53 | <.001 |
| *CEBPA* | 0.3 | 0.02-1.8 | .26 |
| *NRAS* | 0.43 | 0.12-1.2 | .14 |
| *ETV6* | 0.64 | 0.03-5.1 | .7 |
| *RUNX1* | 0.78 | 0.29-1.9 | .6 |
| *GPR98* | 1.1 | 0.22-4.1 | .94 |
| *IDH2* | 1.1 | 0.45-2.6 | .81 |
| *SUZ12* | 1.3 | 0.17-8.2 | .76 |
| *TET2* | 1.3 | 0.62-2.7 | .47 |
| *IDH1* | 1.5 | 0.6-3.5 | .39 |
| *SMC3* | 1.5 | 0.28-6.8 | .62 |
| *PTPN11* | 1.7 | 0.53-5.4 | .35 |
| *PHF6* | 1.9 | 0.51-7 | .33 |
| *CUX1* | 2 | 0.23-17 | .51 |
| *KDM6A* | 2 | 0.23-17 | .51 |
| *NF1* | 2 | 0.36-11 | .41 |
| *APC* | 3.4 | 0.93-14 | .07 |
| *RAD21* | 4.1 | 1.1-20 | .05 |
| *U2AF1* | 5.4 | 1.7-20 | .01 |
| *BCOR* | 7.4 | 1.7-50 | .01 |
| *STAG2* | 7.6 | 2.8-24 | <.001 |
| *EZH2* | 7.7 | 1.1-150 | .07 |
| *BCORL1* | 8.1 | 1.2-160 | .06 |
| *MECOM* | 8.9 | 1.3-180 | .05 |
| *ASXL1* | 9.3 | 2.9-42 | <.001 |
| *CBL* | 9.7 | 1.5-190 | .04 |
| *SF3B1* | 9.7 | 1.5-190 | .04 |
| *SETBP1* | 10 | 1.6-200 | .04 |
| *JAK2* | 12 | 2-230 | .02 |

**Table S 3**: Primary AML vs. secondary AML logistic regression (Intermediate risk cytogenetics , mutations only)

| Gene | OR | CI | P |
| --- | --- | --- | --- |
| *BCORL1* | 10 | 1.8-190 | .03 |
| *EZH2* | 9.6 | 1.7-180 | .04 |
| *SF3B1* | 9.6 | 1.7-180 | .04 |
| *BCOR* | 9.2 | 2.4-60 | <.001 |
| *ASXL1* | 6.5 | 2.7-18 | <.001 |
| *CBL* | 6.2 | 1.6-41 | .02 |
| *STAG2* | 6 | 2.5-17 | <.001 |
| *JAK2* | 5.5 | 1.3-37 | .03 |
| *U2AF1* | 4.3 | 1.8-11 | <.001 |
| *DHX29* | 7.8 | 1.2-150 | .06 |
| *SETBP1* | 7.2 | 1.1-140 | .07 |
| *MECOM* | 6.1 | 0.89-120 | .11 |
| *ZRSR2* | 5.7 | 0.83-110 | .12 |
| *GATA2* | 4.3 | 0.54-87 | .21 |
| *KIT* | 4 | 0.5-81 | .23 |
| *PRPF8* | 3.1 | 0.59-22 | .2 |
| *NF1* | 2.9 | 0.74-14 | .14 |
| *RAD21* | 2.9 | 0.74-14 | .14 |
| *APC* | 2.3 | 0.64-9.2 | .2 |
| *KDM6A* | 2.1 | 0.35-16 | .42 |
| *TP53* | 2 | 0.32-15 | .46 |
| *PHF6* | 1.9 | 0.72-5.5 | .19 |
| *SMC3* | 1.7 | 0.5-6 | .39 |
| *PTPN11* | 1.6 | 0.57-4.8 | .36 |
| *TET2* | 1.5 | 0.83-2.8 | .17 |
| *CUX1* | 1.4 | 0.17-12 | .74 |
| *ETV6* | 1.4 | 0.26-7.7 | .68 |
| *SUZ12* | 1.4 | 0.33-6.1 | .64 |
| *IDH1* | 1.2 | 0.56-2.4 | .67 |
| *RUNX1* | 0.9 | 0.43-1.9 | .78 |
| *GPR98* | 0.85 | 0.22-2.9 | .79 |
| *NRAS* | 0.83 | 0.36-1.8 | .65 |
| *IDH2* | 0.69 | 0.31-1.5 | .35 |
| *KRAS* | 0.51 | 0.07-2.4 | .43 |

**Table S4**: Primary AML vs. secondary AML logistic regression (Complex karyotype , mutations only)

| Gene | OR | CI | P |
| --- | --- | --- | --- |
| *ZRSR2* | 0.23 | 0.01-1.9 | .21 |
| *KRAS* | 0.46 | 0.06-2.9 | .41 |
| *DNMT3A* | 0.56 | 0.15-2 | .37 |
| *TP53* | 0.95 | 0.38-2.4 | .92 |
| *NF1* | 1.1 | 0.18-8.8 | .91 |
| *RUNX1* | 1.9 | 0.38-14 | .46 |
| *JAK2* | 2.2 | 0.28-47 | .49 |
| *PHF6* | 2.2 | 0.28-47 | .49 |
| *BCOR* | 2.3 | 0.28-48 | .48 |
| *PRPF8* | 3.1 | 0.69-21 | .18 |
| *TET2* | 7.7 | 1.4-150 | .06 |

**Table S5**: Primary AML vs. secondary AML logistic regression (Unfavorable risk cytogenetics , mutations only)

| Gene | OR | CI | P |
| --- | --- | --- | --- |
| *U2AF1* | 0.2 | 0.01-1.5 | .17 |
| *FLT3* | 0.77 | 0.13-4.6 | .77 |
| *EZH2* | 1.2 | 0.18-10 | .83 |
| *DNMT3A* | 1.4 | 0.3-7.7 | .66 |
| *KRAS* | 3.6 | 0.48-74 | .27 |
| *SF3B1* | 4 | 0.53-82 | .23 |

**Table S6**: Primary AML vs. secondary AML logistic regression (Age < 60 years , mutations only)

| Gene | Odd Ratio | 95% confidence interval | | *P* |
| --- | --- | --- | --- | --- |
| *FLT3* | 0.067 | 0-0.33 | .01 | |
| *NPM1* | 0.22 | 0.06-0.59 | .01 | |
| *KRAS* | 0.37 | 0.02-2.2 | .36 | |
| *DNMT3A* | 0.4 | 0.14-0.98 | .06 | |
| *NF1* | 0.49 | 0.03-3.2 | .52 | |
| *IDH1* | 0.58 | 0.16-1.7 | .35 | |
| *NRAS* | 0.67 | 0.14-2.3 | .55 | |
| *IDH2* | 0.75 | 0.16-2.6 | .67 | |
| *TET2* | 0.75 | 0.11-3.4 | .73 | |
| *RUNX1* | 0.91 | 0.24-2.9 | .87 | |
| *ZRSR2* | 1.3 | 0.17-6.7 | .79 | |
| *CUX1* | 1.7 | 0.22-11 | .56 | |
| *PHF6* | 1.8 | 0.34-8.2 | .47 | |
| *SUZ12* | 1.8 | 0.35-8.6 | .44 | |
| *PTPN11* | 2.2 | 0.61-7.8 | .2 | |
| *BCORL1* | 2.6 | 0.47-15 | .25 | |
| *GATA2* | 2.6 | 0.3-22 | .35 | |
| *STAG2* | 3.4 | 0.86-14 | .08 | |
| *BCOR* | 4.2 | 1.1-17 | .03 | |
| *TP53* | 5 | 1.3-25 | .03 | |
| *ASXL1* | 7.6 | 1.7-53 | .02 | |
| *SF3B1* | 7.6 | 1.7-53 | .02 | |
| *CBL* | 9.8 | 1.4-190 | .04 | |
| *U2AF1* | 11 | 1.6-220 | .04 | |

**Table S7**: Primary AML vs. secondary AML logistic regression (Age > 60 years , mutations only)

| Gene | Odd Ratio | 95% confidence interval | | *P* |
| --- | --- | --- | --- | --- |
| *NF1* | 8.6 | 1.6-160 | .04 | |
| *ASXL1* | 5.4 | 2.2-16 | <.001 | |
| *PRPF8* | 4.3 | 1.4-19 | .02 | |
| *STAG2* | 3.2 | 1.2-9.8 | .03 | |
| *DHX29* | 7.2 | 1.3-130 | .06 | |
| *SF3B1* | 5.6 | 0.99-110 | .11 | |
| *C7orf55* | 3.5 | 0.51-70 | .26 | |
| *GPR98* | 3.1 | 0.73-21 | .16 | |
| *EZH2* | 2.9 | 0.9-13 | .1 | |
| *CBL* | 2.8 | 0.65-19 | .21 | |
| *KDM6A* | 2.5 | 0.31-50 | .44 | |
| *PTPN11* | 2.5 | 0.56-17 | .27 | |
| *SETBP1* | 2.5 | 0.56-17 | .27 | |
| *JAK2* | 2.2 | 0.73-8.1 | .19 | |
| *SUZ12* | 2.1 | 0.44-15 | .39 | |
| *PHF6* | 2 | 0.65-7.5 | .25 | |
| *WT1* | 2 | 0.42-14 | .42 | |
| *APC* | 1.7 | 0.45-8.4 | .44 | |
| *IDH1* | 1.6 | 0.64-4.3 | .34 | |
| *RAD21* | 1.6 | 0.42-7.9 | .49 | |
| *U2AF1* | 1.5 | 0.72-3.5 | .28 | |
| *SMC3* | 1.4 | 0.42-5.6 | .57 | |
| *TET2* | 1.4 | 0.75-2.5 | .32 | |
| *KRAS* | 1.1 | 0.34-3.7 | .9 | |
| *RUNX1* | 0.98 | 0.48-2 | .96 | |
| *TP53* | 0.88 | 0.41-1.9 | .73 | |
| *CDH23* | 0.85 | 0.1-7.2 | .87 | |
| *CEBPA* | 0.76 | 0.09-6.4 | .79 | |
| *NRAS* | 0.76 | 0.31-1.8 | .54 | |
| *KIT* | 0.38 | 0.05-2 | .26 | |
| *DNMT3A* | 0.34 | 0.17-0.63 | <.001 | |
| *IDH2* | 0.33 | 0.13-0.76 | .01 | |
| *FLT3* | 0.15 | 0.06-0.36 | <.001 | |
| *NPM1* | 0.14 | 0.06-0.31 | <.001 | |

**Table S8**: Primary AML vs. secondary AML logistic regression (WBC > 15.000 , mutations only)

| Gene | OR | CI | P |
| --- | --- | --- | --- |
| *FLT3* | 0.073 | 0.01-0.25 | <.001 |
| *NPM1* | 0.16 | 0.05-0.41 | <.001 |
| *DNMT3A* | 0.2 | 0.06-0.54 | <.001 |
| *WT1* | 0.28 | 0.02-1.6 | .23 |
| *CEBPA* | 0.37 | 0.02-2.3 | .37 |
| *IDH1* | 0.51 | 0.11-1.7 | .31 |
| *U2AF1* | 0.64 | 0.03-4.5 | .69 |
| *GPR98* | 0.67 | 0.03-4.7 | .72 |
| *NRAS* | 0.81 | 0.25-2.3 | .71 |
| *IDH2* | 0.86 | 0.18-3.1 | .83 |
| *PTPN11* | 0.86 | 0.04-6.9 | .9 |
| *KRAS* | 1.2 | 0.16-6.2 | .86 |
| *PHF6* | 1.2 | 0.16-6.2 | .86 |
| *SMC3* | 1.3 | 0.18-7 | .76 |
| *SUZ12* | 2.5 | 0.45-14 | .27 |
| *RAD21* | 2.7 | 0.31-23 | .33 |
| *SETBP1* | 2.7 | 0.31-23 | .33 |
| *APC* | 2.8 | 0.51-16 | .21 |
| *RUNX1* | 3 | 1.1-8.5 | .04 |
| *TET2* | 3.2 | 1.4-7.3 | .01 |
| *NF1* | 3.5 | 0.89-15 | .07 |
| *EZH2* | 6.4 | 1.3-46 | .03 |
| *CBL* | 7.4 | 0.92-150 | .09 |
| *SF3B1* | 7.4 | 0.92-150 | .09 |
| *BCOR* | 8.8 | 1.9-62 | .01 |
| *PRPF8* | 15 | 2.4-300 | .01 |
| *JAK2* | 16 | 2.6-300 | .01 |
| *STAG2* | 18 | 2.9-340 | .01 |

**Table S 9**: Primary AML vs. secondary AML logistic regression (WBC < 15.000 , mutations only)

| Gene | OR | CI | P |
| --- | --- | --- | --- |
| *ASXL1* | 3.3 | 1.5-8.5 | .01 |
| *U2AF1* | 2.6 | 1.2-6.5 | .02 |
| *APC* | 4.5 | 0.71-87 | .17 |
| *DHX29* | 4.5 | 0.71-87 | .17 |
| *BCOR* | 4.4 | 1.1-29 | .06 |
| *SETBP1* | 4.3 | 0.68-82 | .19 |
| *SF3B1* | 4.2 | 1.1-28 | .07 |
| *MECOM* | 3.6 | 0.52-71 | .25 |
| *GATA2* | 3.4 | 0.49-67 | .28 |
| *CBL* | 3.3 | 0.8-22 | .14 |
| *PRPF8* | 3.1 | 0.92-14 | .09 |
| *EZH2* | 2.8 | 0.67-19 | .2 |
| *NF1* | 2.6 | 0.58-18 | .26 |
| *RAD21* | 2.6 | 0.58-18 | .26 |
| *BCORL1* | 2.3 | 0.64-11 | .23 |
| *PHF6* | 2.3 | 0.76-8.4 | .17 |
| *SMC3* | 2.1 | 0.45-15 | .38 |
| *STAG2* | 1.9 | 0.8-4.7 | .16 |
| *PTPN11* | 1.7 | 0.59-5.6 | .34 |
| *GPR98* | 1.5 | 0.36-7.3 | .6 |
| *SUZ12* | 1.4 | 0.34-7 | .64 |
| *TET2* | 1.4 | 0.68-2.8 | .39 |
| *C7orf55* | 1.3 | 0.22-10 | .75 |
| *IDH1* | 1.3 | 0.58-3.3 | .5 |
| *JAK2* | 1.3 | 0.32-6.6 | .7 |
| *ZRSR2* | 1.3 | 0.35-5 | .73 |
| *WT1* | 1.1 | 0.23-5.5 | .93 |
| *NRAS* | 1 | 0.37-3 | .95 |
| *TP53* | 1 | 0.5-2 | 1 |
| *CUX1* | 0.83 | 0.1-7 | .85 |
| *ETV6* | 0.83 | 0.1-7 | .85 |
| *KDM6A* | 0.83 | 0.15-4.5 | .82 |
| *KRAS* | 0.66 | 0.21-2.1 | .47 |
| *RUNX1* | 0.63 | 0.29-1.3 | .22 |
| *KIT* | 0.52 | 0.07-3.2 | .48 |
| *FLT3* | 0.38 | 0.11-1.1 | .08 |
| *DNMT3A* | 0.52 | 0.27-0.99 | .05 |
| *IDH2* | 0.32 | 0.13-0.73 | .01 |
| *NPM1* | 0.26 | 0.1-0.62 | <.001 |

**Table S10**: Primary AML vs. secondary AML Cox regression (All cases , mutations only)

| Gene | HR | CI | P |
| --- | --- | --- | --- |
| *CEBPA* | 0.59 | 0.24-1.42 | .24 |
| *KIT* | 0.64 | 0.24-1.73 | .38 |
| *SUZ12* | 0.7 | 0.35-1.42 | .32 |
| *DDX41* | 0.71 | 0.23-2.2 | .55 |
| *BCORL1* | 0.72 | 0.39-1.36 | .32 |
| *GPR98* | 0.74 | 0.39-1.38 | .34 |
| *IDH2* | 0.78 | 0.52-1.17 | .23 |
| *SF3B1* | 0.83 | 0.44-1.56 | .56 |
| *NPM1* | 0.88 | 0.67-1.17 | .39 |
| *STAG2* | 0.91 | 0.6-1.37 | .64 |
| *CUX1* | 0.95 | 0.42-2.12 | .9 |
| *KRAS* | 0.95 | 0.56-1.62 | .85 |
| *IDH1* | 0.99 | 0.69-1.43 | .96 |
| *MECOM* | 1.02 | 0.45-2.28 | .97 |
| *PHF6* | 1.02 | 0.64-1.64 | .93 |
| *PTPN11* | 1.09 | 0.66-1.8 | .75 |
| *ZRSR2* | 1.11 | 0.59-2.09 | .74 |
| *KDM6A* | 1.15 | 0.54-2.44 | .71 |
| *BCOR* | 1.16 | 0.72-1.87 | .53 |
| *JAK2* | 1.17 | 0.67-2.04 | .58 |
| *DHX29* | 1.18 | 0.52-2.64 | .7 |
| *NF1* | 1.19 | 0.71-2 | .51 |
| *RUNX1* | 1.19 | 0.85-1.66 | .31 |
| *NRAS* | 1.2 | 0.82-1.75 | .35 |
| *GATA2* | 1.21 | 0.57-2.56 | .62 |
| *ETV6* | 1.22 | 0.5-2.95 | .66 |
| *FLT3* | 1.27 | 0.92-1.76 | .14 |
| *WT1* | 1.27 | 0.73-2.21 | .41 |
| *TET2* | 1.31 | 0.99-1.73 | .06 |
| *U2AF1* | 1.37 | 0.96-1.94 | .08 |
| *DNMT3A* | 1.49 | 1.14-1.94 | <.001 |
| *SMC3* | 1.52 | 0.85-2.71 | .16 |
| *APC* | 1.58 | 0.89-2.81 | .12 |
| *RAD21* | 1.58 | 0.86-2.88 | .14 |
| *SETBP1* | 1.59 | 0.82-3.08 | .17 |
| *PRPF8* | 1.69 | 1.02-2.8 | .04 |
| *CDH23* | 1.7 | 0.63-4.57 | .29 |
| *ASXL1* | 1.83 | 1.32-2.53 | <.001 |
| *CBL* | 1.97 | 1.13-3.43 | .02 |
| *C7orf55* | 2 | 0.94-4.28 | .07 |
| *EZH2* | 2.37 | 1.41-3.98 | <.001 |
| *TP53* | 3.05 | 2.14-4.34 | <.001 |

|  |
| --- |

**Table S11**: Primary AML vs. secondary AML Cox regression (Normal Karyotype , mutations only)

| Gene | HR | CI | P |
| --- | --- | --- | --- |
| *CBL* | 2.41 | 1.06-5.47 | .04 |
| *SETBP1* | 2.38 | 1.05-5.4 | .04 |
| *ASXL1* | 2.01 | 1.18-3.44 | .01 |
| *APC* | 2 | 1.05-3.82 | .04 |
| *DNMT3A* | 1.85 | 1.29-2.66 | <.001 |
| *FLT3* | 1.81 | 1.21-2.71 | <.001 |
| *SF3B1* | 2.14 | 0.78-5.82 | .14 |
| *EZH2* | 1.96 | 0.8-4.8 | .14 |
| *MECOM* | 1.66 | 0.61-4.51 | .32 |
| *RAD21* | 1.66 | 0.81-3.38 | .17 |
| *SMC3* | 1.49 | 0.66-3.39 | .34 |
| *U2AF1* | 1.46 | 0.82-2.57 | .2 |
| *WT1* | 1.46 | 0.71-2.98 | .3 |
| *RUNX1* | 1.36 | 0.83-2.22 | .23 |
| *ETV6* | 1.27 | 0.4-3.98 | .69 |
| *KDM6A* | 1.13 | 0.42-3.09 | .81 |
| *NPM1* | 1.12 | 0.8-1.57 | .5 |
| *BCOR* | 1.09 | 0.51-2.32 | .83 |
| *TET2* | 1.09 | 0.73-1.63 | .68 |
| *IDH1* | 1.06 | 0.64-1.75 | .83 |
| *NRAS* | 1.06 | 0.61-1.84 | .84 |
| *IDH2* | 1.03 | 0.64-1.66 | .91 |
| *PTPN11* | 1.03 | 0.54-1.95 | .93 |
| *CEBPA* | 0.96 | 0.35-2.6 | .94 |
| *PHF6* | 0.84 | 0.4-1.8 | .66 |
| *GPR98* | 0.77 | 0.34-1.74 | .53 |
| *STAG2* | 0.75 | 0.43-1.3 | .31 |
| *SUZ12* | 0.75 | 0.24-2.36 | .63 |
| *JAK2* | 0.7 | 0.29-1.71 | .43 |
| *NF1* | 0.66 | 0.24-1.78 | .41 |
| *CUX1* | 0.64 | 0.2-2 | .44 |
| *BCORL1* | 0.5 | 0.16-1.57 | .24 |

**Table S12**: Primary AML vs. secondary AML Cox regression (Intermediate risk cytogenetics , mutations only)

| Gene | HR | CI | P |
| --- | --- | --- | --- |
| *EZH2* | 2.58 | 1.27-5.25 | .01 |
| *CBL* | 2.54 | 1.38-4.68 | <.001 |
| *ASXL1* | 2.06 | 1.39-3.04 | <.001 |
| *DNMT3A* | 1.68 | 1.23-2.31 | .00 |
| *U2AF1* | 1.6 | 1.05-2.43 | .03 |
| *FLT3* | 1.59 | 1.1-2.31 | .01 |
| *SETBP1* | 2.24 | 0.99-5.06 | .05 |
| *SF3B1* | 2.2 | 0.97-4.99 | .06 |
| *TP53* | 2.12 | 0.87-5.19 | .1 |
| *APC* | 1.85 | 0.98-3.51 | .06 |
| *SMC3* | 1.62 | 0.85-3.06 | .14 |
| *KRAS* | 1.59 | 0.75-3.39 | .23 |
| *MECOM* | 1.57 | 0.58-4.24 | .37 |
| *RAD21* | 1.55 | 0.76-3.14 | .23 |
| *ETV6* | 1.5 | 0.62-3.64 | .38 |
| *WT1* | 1.48 | 0.78-2.8 | .23 |
| *GATA2* | 1.33 | 0.49-3.57 | .58 |
| *NRAS* | 1.29 | 0.83-2 | .26 |
| *RUNX1* | 1.29 | 0.86-1.93 | .22 |
| *TET2* | 1.29 | 0.93-1.79 | .13 |
| *KDM6A* | 1.19 | 0.49-2.9 | .71 |
| *PTPN11* | 1.11 | 0.62-1.99 | .72 |
| *SUZ12* | 1.1 | 0.49-2.49 | .81 |
| *IDH1* | 1.07 | 0.71-1.62 | .74 |
| *PHF6* | 1.07 | 0.62-1.84 | .81 |
| *NPM1* | 1.05 | 0.77-1.43 | .75 |
| *JAK2* | 1.01 | 0.5-2.06 | .97 |
| *STAG2* | 1.01 | 0.64-1.58 | .97 |
| *BCOR* | 0.98 | 0.55-1.76 | .96 |
| *ZRSR2* | 0.97 | 0.36-2.62 | .96 |
| *IDH2* | 0.95 | 0.62-1.45 | .81 |
| *NF1* | 0.93 | 0.43-1.97 | .84 |
| *DHX29* | 0.91 | 0.29-2.85 | .87 |
| *PRPF8* | 0.9 | 0.33-2.41 | .83 |
| *KIT* | 0.74 | 0.18-2.97 | .67 |
| *BCORL1* | 0.69 | 0.3-1.55 | .37 |
| *CEBPA* | 0.66 | 0.25-1.79 | .42 |
| *GPR98* | 0.66 | 0.31-1.41 | .29 |
| *CUX1* | 0.59 | 0.19-1.86 | .37 |

**Table S13**: Primary AML vs. secondary AML Cox regression (Complex Karyotype , mutations only)

| Gene | HR | CI | P |
| --- | --- | --- | --- |
| *ASXL1* | 4.03 | 1.58-10.3 | <.001 |
| *TET2* | 2.03 | 1.03-3.98 | .04 |
| *TP53* | 1.98 | 1.21-3.25 | .01 |
| *BCOR* | 2.39 | 0.85-6.73 | .1 |
| *PRPF8* | 1.85 | 0.88-3.88 | .11 |
| *NF1* | 1.72 | 0.69-4.28 | .25 |
| *RUNX1* | 1.47 | 0.64-3.4 | .37 |
| *DNMT3A* | 1.38 | 0.66-2.89 | .39 |
| *JAK2* | 1.1 | 0.4-3.01 | .86 |
| *PHF6* | 1.05 | 0.38-2.89 | .92 |
| *U2AF1* | 0.87 | 0.27-2.77 | .81 |
| *ZRSR2* | 0.76 | 0.28-2.1 | .6 |
| *KRAS* | 0.31 | 0.08-1.27 | .1 |

**Table S14**: Primary AML vs. secondary AML Cox regression (Unfavorable risk cytogenetics, mutations only)

| Gene | HR | CI | P |
| --- | --- | --- | --- |
| *DNMT3A* | 3.45 | 1.47-8.09 | <.001 |
| *EZH2* | 2.27 | 0.79-6.58 | .13 |
| *U2AF1* | 1.47 | 0.56-3.88 | .44 |
| *KRAS* | 1.4 | 0.48-4.06 | .53 |
| *FLT3* | 1.11 | 0.43-2.89 | .83 |
| *SUZ12* | 0.19 | 0.03-1.42 | .11 |
| *SF3B1* | 0.09 | 0.01-0.67 | .02 |

**Table S15**: Primary AML vs. secondary AML Cox regression (Age < 60 years, mutations only)

| Gene | HR | CI | P |
| --- | --- | --- | --- |
| *GPR98* | 0.35 | 0.09-1.44 | .15 |
| *SUZ12* | 0.42 | 0.1-1.7 | .22 |
| *KRAS* | 0.47 | 0.15-1.48 | .2 |
| *SF3B1* | 0.48 | 0.15-1.52 | .22 |
| *CEBPA* | 0.57 | 0.14-2.31 | .43 |
| *TET2* | 0.7 | 0.28-1.72 | .43 |
| *STAG2* | 0.77 | 0.31-1.89 | .57 |
| *BCORL1* | 0.81 | 0.33-1.99 | .64 |
| *IDH1* | 0.85 | 0.46-1.59 | .61 |
| *NPM1* | 0.95 | 0.6-1.5 | .83 |
| *RUNX1* | 0.95 | 0.49-1.84 | .88 |
| *NF1* | 0.99 | 0.4-2.46 | .99 |
| *NRAS* | 1.02 | 0.51-2.02 | .96 |
| *IDH2* | 1.06 | 0.52-2.18 | .88 |
| *ETV6* | 1.15 | 0.28-4.66 | .85 |
| *U2AF1* | 1.19 | 0.49-2.94 | .7 |
| *CUX1* | 1.2 | 0.44-3.27 | .72 |
| *PHF6* | 1.21 | 0.53-2.75 | .65 |
| *PTPN11* | 1.52 | 0.74-3.13 | .26 |
| *WT1* | 1.55 | 0.72-3.37 | .26 |
| *DNMT3A* | 1.59 | 1.01-2.5 | .04 |
| *ZRSR2* | 1.6 | 0.65-3.94 | .31 |
| *FLT3* | 1.64 | 0.99-2.72 | .06 |
| *BCOR* | 1.66 | 0.84-3.3 | .15 |
| *ASXL1* | 2.54 | 1.23-5.25 | .01 |
| *GATA2* | 2.78 | 1.01-7.64 | .05 |
| *CBL* | 3.29 | 1.32-8.19 | .01 |
| *TP53* | 5.39 | 2.65-10.96 | <.001 |

**Table S16**: Primary AML vs. secondary AML Cox regression (Age > 60 years, mutations only)

| Gene | HR | CI | P |
| --- | --- | --- | --- |
| *KIT* | 0.44 | 0.14-1.37 | .16 |
| *IDH2* | 0.61 | 0.38-0.99 | .04 |
| *CEBPA* | 0.79 | 0.25-2.47 | .69 |
| *BCORL1* | 0.81 | 0.33-1.98 | .65 |
| *DHX29* | 0.84 | 0.37-1.89 | .67 |
| *STAG2* | 0.84 | 0.53-1.35 | .47 |
| *JAK2* | 0.85 | 0.48-1.49 | .57 |
| *PHF6* | 0.9 | 0.5-1.61 | .73 |
| *BCOR* | 1 | 0.51-1.96 | .99 |
| *MECOM* | 1.01 | 0.38-2.73 | .98 |
| *NPM1* | 1.01 | 0.7-1.45 | .98 |
| *ZRSR2* | 1.01 | 0.42-2.45 | .98 |
| *RAD21* | 1.05 | 0.52-2.12 | .9 |
| *PTPN11* | 1.06 | 0.52-2.15 | .87 |
| *SUZ12* | 1.08 | 0.48-2.42 | .86 |
| *TET2* | 1.1 | 0.81-1.5 | .53 |
| *U2AF1* | 1.12 | 0.76-1.64 | .58 |
| *GPR98* | 1.17 | 0.58-2.38 | .66 |
| *CDH23* | 1.23 | 0.46-3.31 | .68 |
| *RUNX1* | 1.25 | 0.85-1.85 | .26 |
| *FLT3* | 1.26 | 0.83-1.9 | .28 |
| *APC* | 1.28 | 0.65-2.49 | .47 |
| *SMC3* | 1.31 | 0.7-2.48 | .4 |
| *IDH1* | 1.33 | 0.84-2.1 | .22 |
| *ASXL1* | 1.38 | 0.96-1.99 | .08 |
| *NRAS* | 1.38 | 0.87-2.18 | .18 |
| *NF1* | 1.44 | 0.76-2.72 | .26 |
| *SETBP1* | 1.55 | 0.77-3.15 | .22 |
| *KRAS* | 1.59 | 0.86-2.92 | .14 |
| *CBL* | 1.6 | 0.79-3.24 | .19 |
| *DNMT3A* | 1.63 | 1.17-2.27 | <.001 |
| *WT1* | 1.75 | 0.77-3.96 | .18 |
| *PRPF8* | 1.8 | 1.06-3.04 | .03 |
| *SF3B1* | 1.93 | 0.9-4.12 | .09 |
| *TP53* | 2.24 | 1.49-3.36 | <.001 |
| *EZH2* | 2.46 | 1.43-4.25 | .00 |
| *KDM6A* | 2.49 | 0.92-6.74 | .07 |
| *C7orf55* | 3.6 | 1.47-8.83 | .01 |

**Table S16**: Primary AML vs. secondary AML Cox regression (WBC > 15.000, mutations only)

| *Gene* | HR | CI | P |
| --- | --- | --- | --- |
| *CBL* | 7.44 | 2.61-21.17 | <.001 |
| *RUNX1* | 2.54 | 1.46-4.4 | <.001 |
| *ASXL1* | 2.44 | 1.27-4.69 | .01 |
| *SF3B1* | 2.33 | 0.73-7.39 | .15 |
| *EZH2* | 1.93 | 0.84-4.41 | .12 |
| *JAK2* | 1.78 | 0.83-3.84 | .14 |
| *U2AF1* | 1.53 | 0.62-3.77 | .35 |
| *DNMT3A* | 1.47 | 0.97-2.21 | .07 |
| *BCOR* | 1.45 | 0.7-2.98 | .31 |
| *FLT3* | 1.45 | 0.97-2.16 | .07 |
| *APC* | 1.41 | 0.62-3.21 | .42 |
| *TET2* | 1.31 | 0.83-2.05 | .24 |
| *SUZ12* | 1.3 | 0.48-3.53 | .61 |
| *SETBP1* | 1.22 | 0.39-3.85 | .73 |
| *NF1* | 1.21 | 0.59-2.48 | .61 |
| *NRAS* | 1.18 | 0.68-2.07 | .56 |
| *PRPF8* | 1.1 | 0.4-2.98 | .86 |
| *WT1* | 1.05 | 0.51-2.17 | .89 |
| *SMC3* | 0.93 | 0.38-2.28 | .87 |
| *PTPN11* | 0.84 | 0.31-2.29 | .73 |
| *NPM1* | 0.83 | 0.56-1.22 | .35 |
| *IDH1* | 0.81 | 0.41-1.6 | .55 |
| *IDH2* | 0.78 | 0.38-1.6 | .5 |
| *PHF6* | 0.74 | 0.27-2 | .55 |
| *RAD21* | 0.71 | 0.23-2.25 | .57 |
| *STAG2* | 0.7 | 0.29-1.73 | .45 |
| *CEBPA* | 0.66 | 0.24-1.8 | .42 |
| *GPR98* | 0.62 | 0.2-1.95 | .41 |
| *KRAS* | 0.24 | 0.06-0.98 | .05 |

**Table S17**: Primary AML vs. secondary AML Cox regression (WBC < 15.000, mutations only)

| Gene | HR | CI | P |
| --- | --- | --- | --- |
| *TP53* | 3.32 | 2.26-4.87 | <.001 |
| *RAD21* | 2.79 | 1.37-5.69 | <.001 |
| *EZH2* | 2.68 | 1.37-5.25 | <.001 |
| *SMC3* | 2.5 | 1.17-5.33 | .02 |
| *PRPF8* | 2.19 | 1.22-3.93 | .01 |
| *KRAS* | 1.89 | 1.06-3.39 | .03 |
| *ASXL1* | 1.74 | 1.2-2.55 | <.001 |
| *DNMT3A* | 1.45 | 1.02-2.06 | .04 |
| *SETBP1* | 1.89 | 0.84-4.26 | .13 |
| *APC* | 1.7 | 0.75-3.85 | .2 |
| *WT1* | 1.68 | 0.69-4.1 | .25 |
| *ETV6* | 1.64 | 0.52-5.13 | .4 |
| *CBL* | 1.52 | 0.78-2.96 | .22 |
| *C7orf55* | 1.46 | 0.59-3.6 | .41 |
| *GATA2* | 1.45 | 0.6-3.54 | .41 |
| *U2AF1* | 1.37 | 0.93-2.01 | .11 |
| *CUX1* | 1.32 | 0.49-3.55 | .58 |
| *TET2* | 1.28 | 0.89-1.84 | .18 |
| *NRAS* | 1.23 | 0.73-2.07 | .45 |
| *PTPN11* | 1.23 | 0.69-2.2 | .49 |
| *DHX29* | 1.18 | 0.44-3.19 | .74 |
| *PHF6* | 1.14 | 0.67-1.96 | .63 |
| *NF1* | 1.08 | 0.51-2.31 | .84 |
| *ZRSR2* | 1.06 | 0.54-2.06 | .87 |
| *IDH1* | 1.04 | 0.67-1.62 | .85 |
| *STAG2* | 1.01 | 0.63-1.62 | .96 |
| *KDM6A* | 0.99 | 0.44-2.23 | .97 |
| *MECOM* | 0.99 | 0.37-2.67 | .99 |
| *BCOR* | 0.95 | 0.5-1.79 | .87 |
| *FLT3* | 0.86 | 0.44-1.67 | .65 |
| *NPM1* | 0.86 | 0.54-1.36 | .52 |
| *RUNX1* | 0.84 | 0.55-1.29 | .44 |
| *JAK2* | 0.81 | 0.36-1.82 | .61 |
| *BCORL1* | 0.8 | 0.42-1.51 | .49 |
| *IDH2* | 0.8 | 0.49-1.29 | .35 |
| *GPR98* | 0.78 | 0.35-1.77 | .56 |
| *SF3B1* | 0.64 | 0.3-1.36 | .24 |
| *KIT* | 0.49 | 0.12-1.99 | .32 |
| *SUZ12* | 0.47 | 0.17-1.26 | .13 |
|  |  |  |  |
|  |  |  |  |

**Table S18**: Primary versus secondary AML multivariate Cox model analysis (Including treatment intensity)

| Parameter | Hazard ratio | 95% Confidence interval | *P* |
| --- | --- | --- | --- |
| Diagnosis  (Primary vs. Secondary AML) | 1.11 | 0.78 – 1.56 | .06 |
| Age  ( < 60 vs. > 60) | 1.73 | 1.24 – 2.41 | <.001 |
| Treatment  (Intensive vs. Non-intensive) | 1.35 | 0.99 – 1.83 | .06 |
| Cytogenetic analysis  (Normal Karyotype vs. Others) | 1.12 | 0.8 - 1.57 | .51 |
| Cytogenetic analysis  (Normal Karyotype vs. Others) | 3.04 | 2.04 - 4.51 | <.001 |
| Cytogenetic analysis  (Normal Karyotype vs. Others) | 1.76 | 1.09 - 2.84 | .02 |
| WBC  (</= 15,000 vs. > 15,000) | 1.5 | 1.1 – 2.06 | .01 |
| *DNMT3A* | 1.59 | 1.06 - 2.38 | .02 |
| *ASXL1* | 1.84 | 1.22 - 2.77 | <.001 |
| *EZH2* | 2.56 | 1.32 – 4.97 | <.001 |
| *TP53* | 2.09 | 1.29 - 3.41 | <.001 |
